# Economic burden of cancer care in Lebanon’s privately insured population: a seven-year retrospective cohort analysis (2018–2024)

**DOI:** 10.3389/fonc.2026.1924237

**Published:** 2026-08-25

**Authors:** Shadi Saleh, Paul Makhoul, Rawad Chamandi, Zahi Abdul-Sater

**Affiliations:** 1 Department of Health Management and Policy, Faculty of Health Sciences, American University of Beirut, Beirut, Lebanon; 2 Global Health Institute, American University of Beirut, Beirut, Lebanon; 3 Market Access, AstraZeneca, Beirut, Lebanon; 4 College of Public Health, Phoenicia University, Mazraat El Daoudiyeh, Lebanon

**Keywords:** cancer care costs, claims analysis, economic burden, high-cost cohort, Lebanon, PMPY, private health insurance, retrospective cohort

## Abstract

**Background:**

Cancer imposes a growing economic burden on health systems globally, yet longitudinal, population-level data on cancer care costs within private insurance remain scarce in low- and middle-income countries. Lebanon’s private insurance sector, administered through third-party administrators (TPAs), covers a substantial share of the economically active population, yet no published study has characterized its cancer-related economic burden, including during the country’s economic contraction since 2019.

**Methods:**

A retrospective cohort analysis used event-level insurance claims from a leading Lebanese TPA for 2018–2024. Claims carrying an ICD-10 cancer diagnosis code (C or D series, excluding benign neoplasms D10–D36) were processed through a standardized pipeline from event to patient level. Analyses covered demographics, cancer type distribution, treatment and service utilization, cost trends, per-member-per-year (PMPY) expenditure, and a high-cost cohort (HCC) defined as USD 100,000 or more in cumulative costs within any rolling 365-day window.

**Results:**

The cohort comprised 16,617 unique cancer patients generating 157,550 claims and USD 239.9 million in total expenditure over seven years. Females accounted for 53.7% of patients; breast cancer was the most prevalent diagnosis (19.7%), in the predominant 40–64-year age group (50.2%). Surgery was the most common treatment modality (29.3%), followed by cancer medicines (24.2%) and radiotherapy (14.9%), yet cancer medicines were the largest cost category (37.8% of spend). Annual expenditure rose from USD 35.7 million (2018) to USD 48.9 million (2024), with a transient compression in 2022. A high-cost cohort of 285 patients (1.7%) accounted for USD 55.1 million (23.0% of spend), led by lung cancer (19.7%). Cancer patient rates rose 24.5% (1,015 to 1,263 per 100,000), and per-member cost rose steeply with age, reaching USD 606 PMPY among patients aged 65+ in 2024 versus USD 15–169 in younger groups; overall PMPY reached USD 111.50, recovering from the 2022 trough.

**Conclusions:**

This study provides the first longitudinal, claims-based characterization of cancer care’s economic burden in Lebanon’s privately insured population. Costs are concentrated in a small, identifiable high-cost subgroup, cancer medicines dominate expenditure despite surgery leading by patient volume, and the per-member burden is rising — an actuarial foundation for designing sustainable, oncology-specific benefit structures.

## 1 Introduction

In 2022, an estimated 20.0 million new cancer cases were diagnosed and 9.7 million cancer-related deaths recorded globally, making cancer the second leading cause of mortality worldwide (1, 2). Annual incidence is projected to reach 35.3 million cases by 2050, a 77% increase driven by population ageing, growth, and ongoing epidemiological transitions in diet, physical activity, and reproductive behavior (1). The economic consequences are commensurate with this scale. The global attributable economic cost of cancer has been estimated at USD 1.16 trillion annually, a figure that excludes the direct medical expenditure on diagnosis, treatment, and supportive care that constitutes the primary burden for health insurers and governments (3, 4). When healthcare costs are included, oncology consistently ranks as the highest-expenditure therapeutic area in national health accounts. Global oncology drug spending alone is projected to exceed USD 370 billion annually by 2027 (5, 7, 8). For private health insurers, cancer is therefore the defining actuarial challenge of the coming decade, simultaneously the most prevalent high-cost condition and the one most sensitive to advances in expensive systemic therapy (6).

The burden is not equitably distributed across income settings. Low- and middle-income countries (LMICs) currently account for approximately 57% of new global cancer diagnoses and nearly 70% of cancer-related deaths yet receive fewer than 20 cents of every dollar spent on cancer care worldwide (9, 10). This financing deficit has clinical consequences. Indeed, access to curative surgery, evidence-based systemic therapy, and radiotherapy remains severely restricted across LMIC settings (32). Additionally, late-stage presentation is the norm rather than the exception, and out-of-pocket cancer expenditure constitutes a leading cause of catastrophic household financial loss (11, 12). Within LMICs, private health insurance administered through third-party administrators (TPAs) has emerged as an increasingly important payer for high-cost specialized care, particularly in countries with fragmented public coverage. Yet the cancer-related economic burden within these schemes, which include the expenditure they bear, the treatment patterns of their insured populations, and the actuarial risk they carry, remains almost entirely unquantified, constituting a critical gap for benefit design, premium setting, and long-term financial sustainability (6, 12).

Lebanon is a small lower-middle-income country in the Eastern Mediterranean that carries a disproportionately high cancer burden relative to its regional peers. GLOBOCAN 2022 estimates Lebanon’s age-standardized cancer incidence rate at 193.7 per 100,000 population, substantially exceeding the Eastern Mediterranean regional average (1). The country’s national cancer registry documents rising trends in incidence across multiple tumor types over the past decade (13). Breast cancer is the leading malignancy among Lebanese women, accounting for approximately 34% of female cancer diagnoses, reflecting the influence of reproductive transitions, dietary shifts, and urbanization (14–16). Colorectal, lung, prostate, and hematological malignancies constitute the other major burden categories, and historically limited population-level cancer screening has contributed to a pattern of late-stage presentation that drives elevated per-patient costs (13, 15). Lebanon thus presents a high-burden, data-poor setting in which population-level evidence on cancer care costs is urgently needed for health system planning and insurance market governance.

Lebanon’s health financing architecture is characterized by deep structural fragmentation. The Ministry of Public Health provides residual coverage for the uninsured population, the National Social Security Fund (NSSF) covers formally employed workers, and a private insurance sector administered through TPAs covers a substantial additional segment. Regulatory filings to the Insurance Control Commission record approximately 627,000 covered lives under private health insurance in 2022, rising to 773,000 by 2024 (17, 18, 34). This TPA sector, operating under the oversight of the Association of Lebanese Insurance Companies (ACAL), administers the majority of financing for high-cost specialized care in Lebanon, including oncology services such as systemic therapy, surgical oncology, and radiotherapy. Despite this structural centrality, no published study has characterized the cancer-related expenditure administered by the Lebanese TPA sector, the treatment patterns of its insured cancer patients, or the per-member actuarial exposure it carries across time. This evidence gap directly constrains the ability of insurers, employers, and policymakers to design evidence-based oncology benefit structures, set risk-appropriate premiums, and target cost management programs at the highest-leverage patient subgroups.

The seven-year study period (2018-2024) coincides with one of the most severe economic crises in modern history. Lebanon’s real GDP contracted by an estimated 58% in US dollar terms between 2018 and 2021, a collapse the World Bank ranked among the world’s three most severe economic crises since the 1850s (19). The Lebanese pound, officially pegged at 1,507 LBP per USD since 1997, collapsed to over 90,000 LBP per USD on the parallel market by 2023, with the formal banking system imposing restrictions on dollar withdrawals and transfers. Health system consequences were immediate and severe. Widespread shortages affected essential and chronic-disease medicines, with the removal of subsidies in late 2021 driving steep price increases and documented unavailability across multiple therapeutic classes, including anticancer agents (35–37). The Lebanese Order of Physicians estimates that approximately 3,500 physicians left the country between 2019 and 2023, alongside substantial nursing attrition (38, 39). Additionally, hospital operating costs escalated sharply as pharmaceuticals and consumables repriced in US dollars, and insurance claims denominated in Lebanese pounds underwent acute USD-equivalent compression (19, 20). Understanding cancer care costs across this extraordinary period, in USD-equivalent terms, and in the context of recovering demand and rising systemic therapy utilization, is therefore essential for actuarial planning, reinsurance structuring, and benefit design for the post-crisis insurance market.

Against this backdrop, the present study uses event-level insurance claims data from a leading Lebanese TPA for 2018–2024 to provide the first longitudinal, claims-based characterization of the economic burden of cancer care in Lebanon’s privately insured population. Specifically, this study aims to: (1) describe the demographic and clinical characteristics of insured cancer patients across the seven-year study period; (2) quantify treatment modality utilization patterns and care setting distribution; (3) characterize total and cancer-type-specific expenditure, identify a high-cost patient subgroup using a rolling 12-month cost threshold, and delineate the drivers of expenditure concentration; (4) assess whether cancer patient rates in the insured population are rising after adjustment for annual enrolment fluctuations; and (5) derive annual cancer expenditure per insured member (per-member-per-year, PMPY) estimates stratified by year, sex, age group, and cancer type to provide an empirical actuarial foundation for benefit design and premium setting in Lebanon’s private insurance sector.

## 2 Methods

### 2.1 Data source and study design

A retrospective cohort analysis was conducted using event-level insurance claims data extracted from a single leading Lebanese TPA for calendar years 2018 to 2024 inclusive. The TPA administers private health insurance plans on behalf of multiple insurance companies and employer groups, covering employees and their dependents across Lebanon. Annual insured population denominators, stratified by sex and age group, were obtained from the TPA. The study was conducted on routinely collected, de-identified administrative data and did not involve primary data collection from human participants. Reporting followed the RECORD statement (21).

### 2.2 Patient identification and cancer classification

All claims carrying at least one ICD-10 diagnosis code in the C (malignant neoplasms, C00–C97) or D (*in situ*, uncertain, and unspecified behavior neoplasms, D00–D09, D37–D49) series were flagged as cancer-related. Benign neoplasms (D10–D36) were excluded. Unique cancer patients were identified by deidentified beneficiary ID and patients appearing in multiple calendar years were counted once across the study period. Of the 16,617 patients, 12,039 (72.4%) were identified through malignant C-series codes only, 3,219 (19.4%) through both C- and D-series codes, and 1,262 (7.6%) through D-series codes only, representing *in situ* and uncertain or unspecified behavior neoplasms. The remaining 97 (0.6%) carried a cancer diagnosis that did not map to the classification taxonomy.

Cancer types were classified using an 82-category ICD-10 taxonomy mapped from three-character code roots (e.g., C50 → Breast; C34 → Lung; C91–C95 → Leukemia). A primary cancer type was assigned to each beneficiary based on their earliest recorded ICD-10 cancer code chronologically, applying a priority hierarchy of malignant (C codes) > *in situ* (D00–D09) > uncertain behavior (D37–D49). This primary attribution was used for all cost analyses to ensure non-overlapping assignment and prevent double-counting. Where a secondary or non-specific code was the earliest recorded, this rule assigns that category, and 525 patients (3.2%) were classified to a secondary, ill-defined or unspecified category on this basis. For cancer-type prevalence analyses, each patient’s complete all-time set of cancer diagnoses was used (patient-level view), and a cancer-level view reported each patient’s contribution once per unique cancer type across their 23,836 total patient–cancer-type assignments.

### 2.3 Data processing pipeline

Raw event-level claims files for each calendar year were processed through a standardized Python preprocessing pipeline. Steps included: (1) removal of exact duplicate rows across all columns, (2) cleaning of length-of-stay values (fractional and negative values coerced to one day), (3) standardization of claim cost fields by stripping currency symbols, removing thousands separators, and converting to numeric, (4) left-join of all claim events to an Item Mapping Dictionary assigning each billed item to one of seven mutually exclusive primary clinical buckets: surgery main, rt_main, chemo_drug, chemo_admin, imaging, ICU, and other; (5) aggregation from event level to claim level; and (6) aggregation from claim level to patient-year level by beneficiary ID.

### 2.4 Treatment modality classification

Treatment modalities were defined using event-level flags derived from the Item Mapping Dictionary, which was developed through systematic manual classification of all distinct billed item names in the dataset. Surgery was defined as the presence of at least one event classified as surgery (major operative procedures, excluding anesthesia lines, operating room assistant fees, and pathology lines). Radiotherapy was defined by radiotherapy events (radiotherapy delivery sessions, excluding planning and simulation). Cancer medicines (systemic therapy) encompassed both medicine related events (antineoplastic drugs classified by substance) and chemotherapy administration events (chemotherapy administration and infusion services). Imaging was defined by imaging events (CT, MRI, ultrasound, X-ray, and selected diagnostic modalities). ICU exposure was derived from ICU bed events in the dictionary and emergency room (ER) exposure from the ER flag on claims. Treatment modality flags were cumulative across all years a patient appeared in the dataset, meaning any single qualifying event in any year was sufficient to classify a patient as having received that modality. Modality categories are therefore not mutually exclusive, apply to the full 2018–2024 period, and do not represent annual treatment rates. Because many Lebanese hospitals submit claims as lump-sum all-inclusive bundles in which individual services are not itemized, modality can be assigned only for claims with itemized service lines.

### 2.5 High-cost cohort definition

The high-cost cohort (HCC) was defined as patients incurring USD 100,000 or more in cumulative insurance expenditure within any rolling 365-day window. Identification used a two-pointer sliding window algorithm applied over temporally ordered claim dates. A hybrid date assignment was employed: the first claim per patient was assigned its earliest transaction date (marking system entry) and all subsequent claims were assigned their latest transaction date (marking when cost was finalized). A rolling window was used in preference to the calendar year so that a treatment episode spanning a year boundary is not artificially divided. To assess whether cohort membership depended on the date convention, identification was repeated using earliest transaction dates throughout and latest transaction dates throughout, and the cohort was identical under all three conventions. Expenditure reported for the high-cost cohort represents the total cost of care for those patients across the full study period rather than cost confined to the qualifying window.

### 2.6 Population analytics, cancer patient rates, and PMPY expenditures

Crude cancer patient rates were expressed per 100,000 insured members, computed as the number of cancer patients appearing in claims in each calendar year divided by the total insured population in that year. Age-standardized rates were computed by applying reference population weights derived from the 2022 insured beneficiary age–sex distribution to year-specific age-group-level crude rates, enabling trend comparison across years with different enrolment profiles. Per-member-per-year (PMPY) cancer expenditure was calculated as total annual cancer claims cost divided by the total insured population for that year, expressed in USD. PMPY was further stratified by sex, age group (0–17, 18–39, 40–64, 65+ years), and primary cancer type. All cost figures are reported in USD, reflecting the TPA’s billing currency; claims are billed and settled in USD. The 2022 expenditure compression reported throughout reflects the acute loss in USD-equivalent value of LBP-denominated cost components during Lebanon’s currency devaluation and Sayrafa exchange-rate transition, rather than a reduction in clinical volume.

## 3 Results

### 3.1 Study population characteristics and cancer patient rate trends

**Figure 1:**
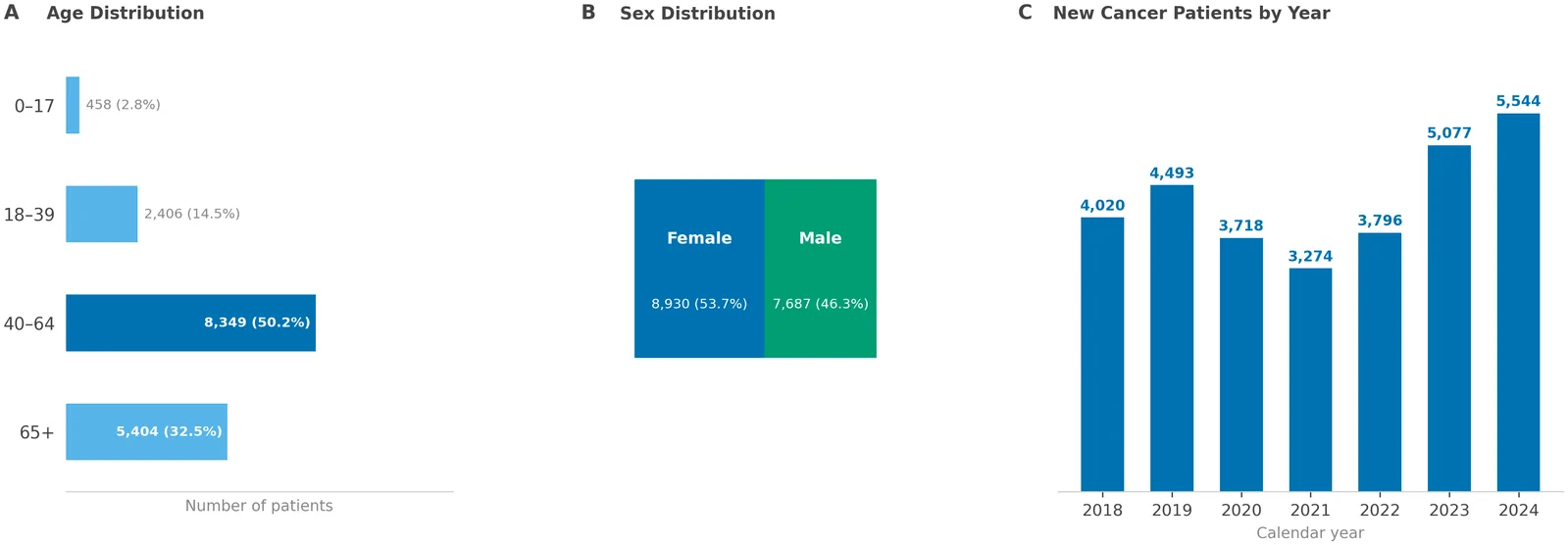
Study cohort demographics (2018–2024). Panel **(A)** Age group distribution of the 16,617 unique cancer patients, shown as counts and proportions for four groups (0–17, 18–39, 40–64, 65+). The 40–64 group predominates (50.2%). Panel **(B)** Sex distribution by calendar year; females consistently represent 53.7% of the cohort, consistent with the predominance of breast cancer. Panel **(C)** Claims-based cancer patient rate by calendar year (left axis, bars) overlaid on the insured population trend (right axis, line), illustrating growth from 4,020 patients in 2018 to 5,544 in 2024 and the mid-period decline during 2020–2021.

The analytic cohort comprised 16,617 unique cancer patients identified from 157,550 claims generating USD 239,870,103 in total expenditure across the seven-year study period (**Table 1**, **Figure 1****).** Annual cancer patient volume grew from 4,020 in 2018 to 5,544 in 2024, with a mid-period decline in 2020 and 2021. Female patients accounted for 53.7% of the cohort (n = 8,930) and males for 46.3% (n = 7,687), consistent with the predominance of breast cancer in the female population. The majority of patients were aged 40–64 years (50.2%, n = 8,349), followed by 65 years and older (32.5%, n = 5,404), 18–39 years (14.5%, n = 2,406), and 0–17 years (2.8%, n = 458). At last follow-up, 15,415 patients (92.8%) were recorded as alive and 1,202 (7.2%) as deceased.

**Table 1:** Study Population Characteristics.

Characteristic	n	%
Study Overview		
Study period	2018–2024	7 years
Total unique cancer patients	16,617	100.0%
Total cancer claims	157,550	—
Total insured population, 2018 (baseline)	396,230	—
Total cancer care expenditure (USD)	$239,870,103	—
Cancer Patients by Calendar Year		
2018	4,020	24.2%
2019	4,493	27.0%
2020	3,718	22.4%
2021	3,274	19.7%
2022	3,796	22.8%
2023	5,077	30.6%
2024	5,544	33.4%
Sex		
Female	8,930	53.7%
Male	7,687	46.3%
Age at Diagnosis		
0–17	458	2.8%
18–39	2,406	14.5%
40–64	8,349	50.2%
65+	5,404	32.5%
Survival Status		
Alive	15,415	92.8%
Deceased	1,202	7.2%

**Figure 2:**
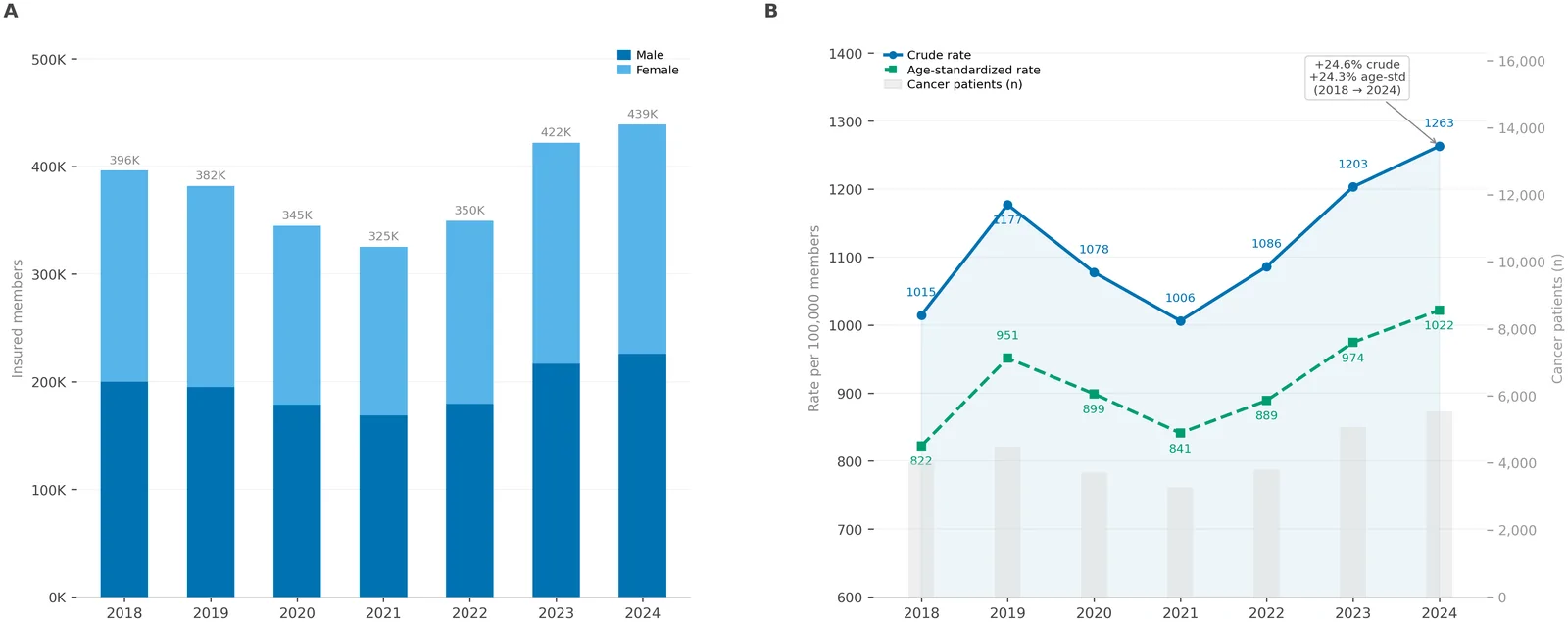
Cancer patient rates in the insured population (2018–2024). Panel **(A)** Annual insured population by year, stacked by sex (male and female), sourced from the TPA beneficiary enrollment file. Population declined from 396,230 in 2018 to a trough of 325,387 in 2021, recovering to 438,911 in 2024. Panel **(B)** Crude cancer patient rate per 100,000 insured members (solid line, left axis) and age-standardized rate (dashed line, left axis) by year, with annual cancer patient counts shown as gray bars (right axis). Crude rate rose from 1,014.6 to 1,263.1 per 100,000 members over the study period (24.5% increase); age-standardized rate from 822.1 to 1,021.9 per 100,000. Age standardization used the 2022 insured beneficiary age–sex distribution as the reference population. Rates reflect cancer patients appearing in claims per year per 100,000 insured members.

The total insured population fluctuated across the study period, declining from 396,230 in 2018 to 325,387 in 2021 before recovering to 438,911 in 2024 (**Figure 2A**). Despite this variable denominator, crude cancer patient rates per 100,000 insured members showed a clear upward trend starting with 1,014.6 per 100,000 in 2018, rising to 1,263.1 per 100,000 in 2024, constituting a 24.5% increase over the study period (**Figure 2B**; **Table 2**). Female rates exceeded male rates throughout the study period, consistent with the predominance of breast cancer in the cohort.

**Table 2:** Annual cancer patient rates in the insured population (2018–2024).

Year	Insured population	Cancer patients (annual)	Rate per 100,000
2018	396,230	4,020	1014.6
2019	381,716	4,493	1177.1
2020	345,039	3,718	1077.6
2021	325,387	3,274	1006.2
2022	349,565	3,796	1085.9
2023	421,945	5,077	1203.2
2024	438,911	5,544	1263.1
**2018–2024**	**—**	16,617*	**—**

Annual counts are not mutually exclusive; patients appearing in multiple calendar years are counted in each year. Total unique patients across all years = 16,617.

Asterisk denotes the total number of unique patients across the full study period, which is not the sum of the annual counts because patients appearing in multiple years are counted once. Bold values indicate study-period totals rather than annual figures.

Age-standardized cancer patient rates, using the 2022 insured beneficiary age–sex distribution as the reference population, mirrored this trend, starting with 822.1 per 100,000 in 2018 and rising to 1,021.9 per 100,000 in 2024, suggesting that the increase was not fully explained by changes in the age–sex composition of the insured pool, though additional factors including evolving care-seeking patterns, screening uptake, and claims capture may also contribute (**Figure 2B**). Age-specific rates demonstrated the steepest absolute values in older age groups: 2,037 per 100,000 members in the 40–64 group and 6,252 per 100,000 in the 65+ group in 2024, compared with 399 per 100,000 in the 18–39 group and 105 per 100,000 in the 0–17 group.

### 3.2 Cancer type distribution

**Figure 3:**
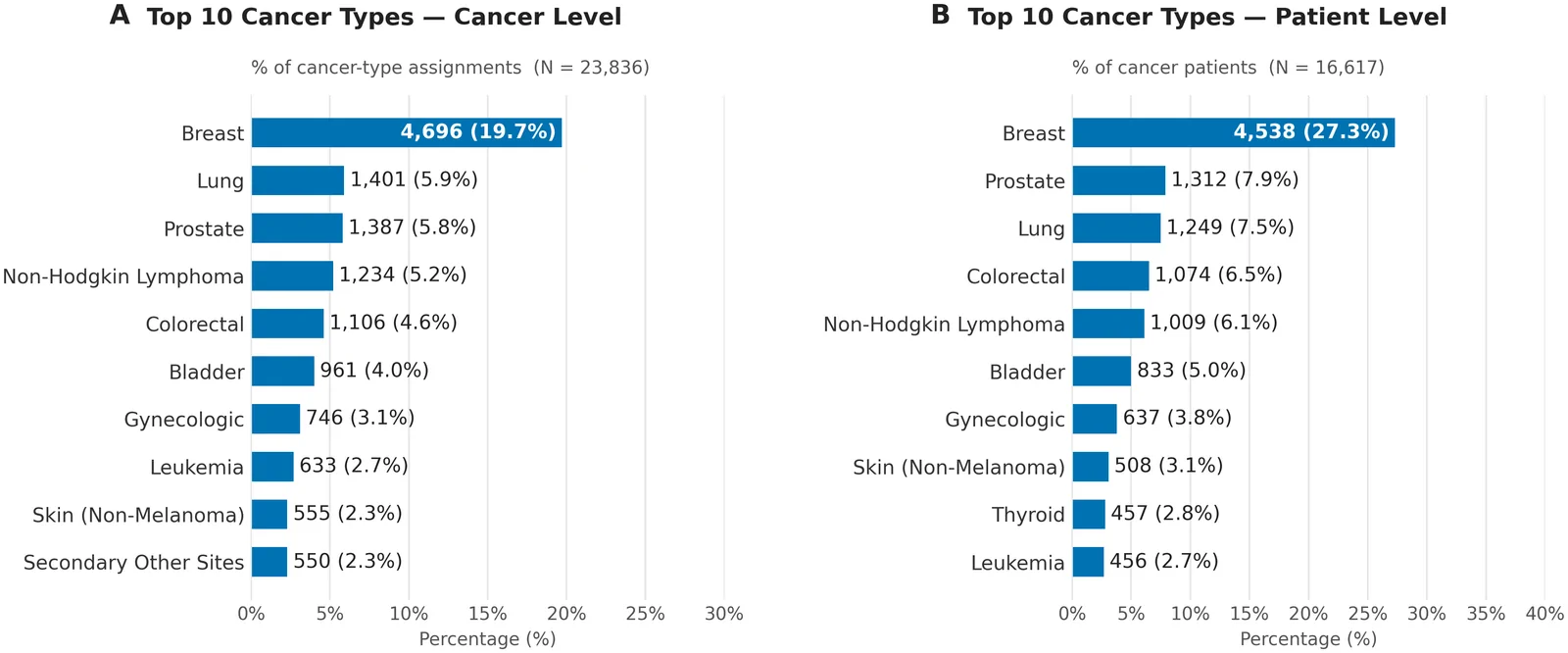
Cancer type distribution) cancer-level and patient-level views (2018–2024). Panel **(A)** Top 10 cancer types by cancer-level prevalence, expressed as percentage of 23,836 all-time patient–cancer-type assignments. Each patient contributes once per unique cancer type in their all-time diagnosis set; patients with multiple cancer types appear in multiple rows. Breast leads at 19.7%. Panel **(B)** Top 10 cancer types by patient-level prevalence, expressed as percentage of 16,617 unique patients; each patient is counted once regardless of the number of cancer types. Breast cancer leads at 27.3%, followed by prostate (7.9%), lung (7.5%), colorectal (6.5%), and non-Hodgkin lymphoma (6.1%). The full 69-category distribution is provided in **Supplementary Table 1**.

Cancer type distribution was characterized at two complementary levels (**Figure 3**; **Table 3**). At the cancer level (proportion of 23,836 all-time patient–cancer-type assignments), breast cancer accounted for 19.7% (n = 4,696), followed by lung (5.9%), prostate (5.8%), non-Hodgkin lymphoma (5.2%), and colorectal (4.6%). The top ten cancer types together accounted for approximately 55% of all cancer-type assignments. At the patient level (proportion of 16,617 unique patients), breast cancer was the most frequent diagnosis, affecting 4,538 patients (27.3%), followed by prostate cancer (1,312 patients, 7.9%), lung cancer (1,249 patients, 7.5%), colorectal cancer (1,074 patients, 6.5%), and non-Hodgkin lymphoma (1,009 patients, 6.1%). The two views rank differently because patients with lung cancer more frequently carry more than one cancer-type assignment across the study period. The complete distribution across all 69 categories is reported in **Supplementary Table 1**.

**Table 3:** Distribution of cancer types — Top 20 (2018–2024).

Rank	Cancer type	Cancer-level assignments (n)	Cancer level %	Unique patients (n)	Patient level %
1	Breast	4,696	19.7%	4,538	27.3%
2	Lung	1,401	5.9%	1,249	7.5%
3	Prostate	1,387	5.8%	1,312	7.9%
4	Non-Hodgkin Lymphoma	1,234	5.2%	1,009	6.1%
5	Colorectal	1,106	4.6%	1,074	6.5%
6	Bladder	961	4.0%	833	5.0%
7	Gynecologic	746	3.1%	637	3.8%
8	Leukemia	633	2.7%	456	2.7%
9	Skin (Non-Melanoma)	555	2.3%	508	3.1%
10	Secondary Other Sites	550	2.3%	56	0.3%
11	Brain & CNS	543	2.3%	386	2.3%
12	Thyroid	486	2.0%	457	2.7%
13	Multiple Myeloma	476	2.0%	410	2.5%
14	Secondary Respiratory/Digestive	460	1.9%	46	0.3%
15	Other Hematopoietic	446	1.9%	392	2.4%
16	Head & Neck	444	1.9%	442	2.7%
17	Hodgkin Lymphoma	429	1.8%	370	2.2%
18	Ill-defined Sites	387	1.6%	243	1.5%
19	Pancreatic	369	1.5%	307	1.8%
20	Unspecified Site	367	1.5%	151	0.9%
	**All 69 cancer types (total)**	**23,836**	**100.0%**	**16,617**	**—**

Unique patient counts reflect the number of patients with at least one claim for that cancer type. Column sums exceed total unique patients (16,617) because patients with multiple cancer diagnoses appear in more than one row. Percentages are calculated against the total of 16,617 unique patients. Bold values in the final row are study-period totals. The cancer-level column sums to 23,836 assignments across all 69 categories, whereas 16,617 is the total number of unique patients and is not the sum of the unique-patient column, because patients with more than one cancer type appear in multiple rows.

### 3.3 Treatment patterns and service utilization

**Figure 4:**
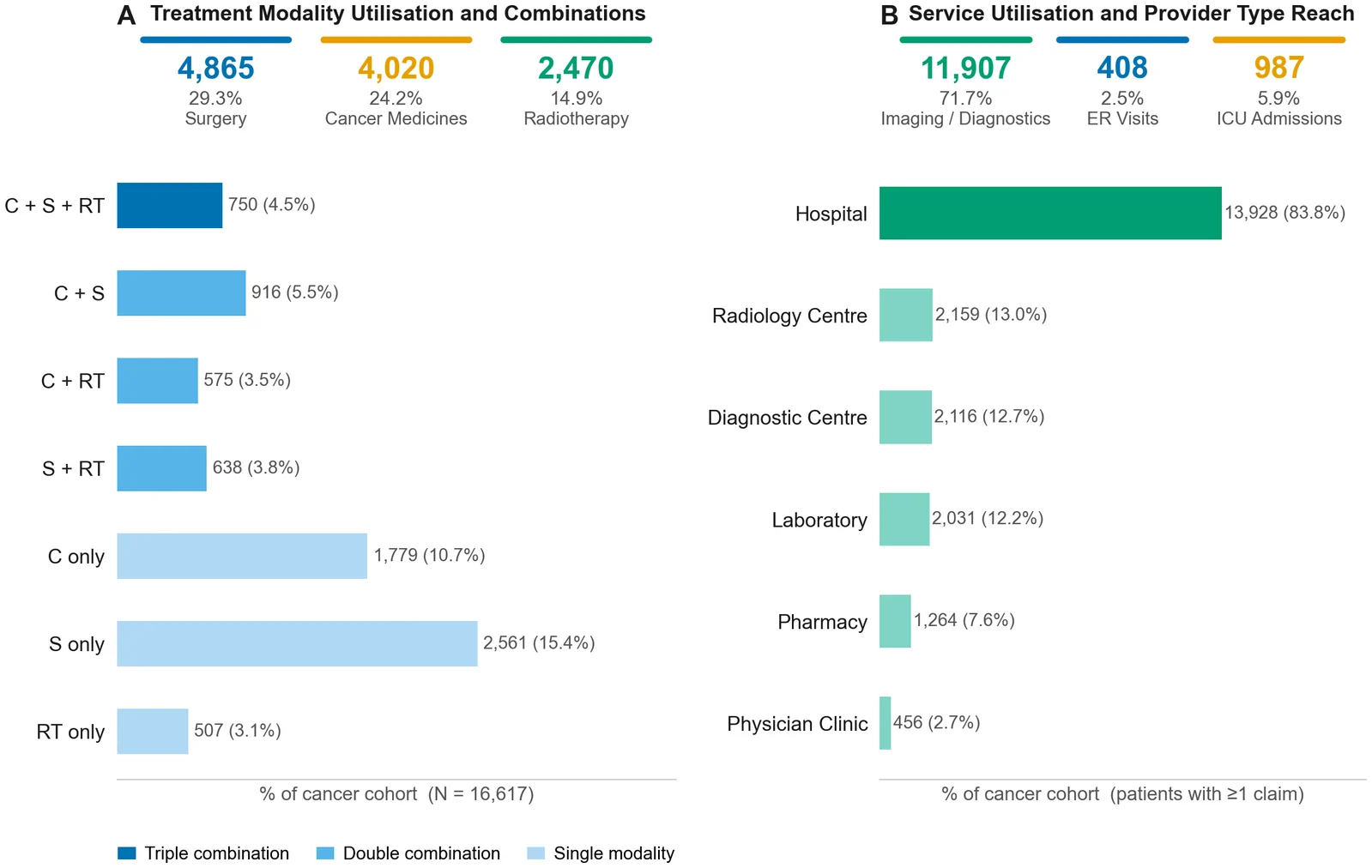
Treatment modality utilization and service patterns (2018–2024). Panel **(A)** Individual modality utilization rates (% of 16,617 patients) for surgery (29.3%), cancer medicines (24.2%), and radiotherapy (14.9%), alongside modality combination breakdown. Surgery alone was the most common single-modality pattern (15.4%); all three modalities combined were received by 4.5% of patients. Panel **(B)** Provider type reach, defined as the proportion of patients with ≥1 claim in each provider category, demonstrating hospital dominance (83.8% of patients). Panel **(C)** Care setting split (inpatient 61.0% vs outpatient 39.0% of hospital claims) and utilization rates for imaging (71.7% of patients), ICU (5.9%), and ER (2.5%).

Surgery was the most frequently received treatment modality, with 4,865 patients (29.3%) having at least one major surgical procedure on record (**Figure 4**). Cancer medicines (systemic therapy, encompassing chemotherapy drugs, immunotherapy agents, and infusion administration) were received by 4,020 patients (24.2%), and radiotherapy by 2,470 patients (14.9%). Imaging services were recorded for 11,907 patients (71.7%), reflecting the near-universal use of diagnostic imaging in oncology care. Modality combination analysis revealed that surgery alone was the most common single-modality pattern (2,561 patients, 15.4%), followed by cancer medicines alone (1,779 patients, 10.7%), cancer medicines and surgery (916, 5.5%), all three modalities (750 patients, 4.5%), surgery and radiotherapy (638, 3.8%), cancer medicines and radiotherapy (575, 3.5%), and radiotherapy alone (507, 3.1%). Together, 7,726 patients (46.5%) had at least one itemized treatment claim, and among these 63.0% received surgery, 52.0% cancer medicines and 32.0% radiotherapy. The remaining 8,891 patients (53.5%) had no itemized record of surgery, radiotherapy or systemic therapy. This group should not be interpreted as untreated. Of these, 5,466 had imaging records only and 3,425 had no itemized clinical event of any kind. Together they accounted for 6.3% of total expenditure, with a median cost of USD 302 per patient, a pattern consistent with all-inclusive bundled billing rather than absence of treatment. Service utilization was dominated by hospital-based care with hospital provider claims comprising 133,362 of all claims (84.6%). Of all claims, 96,158 (61.0%) were inpatient and 61,392 (39.0%) outpatient. ICU admissions were recorded for 987 patients (5.9%) and emergency room visits for 408 patients (2.5%).

### 3.4 Total cancer expenditure, cost drivers, and per-member burden

**Figure 5:**
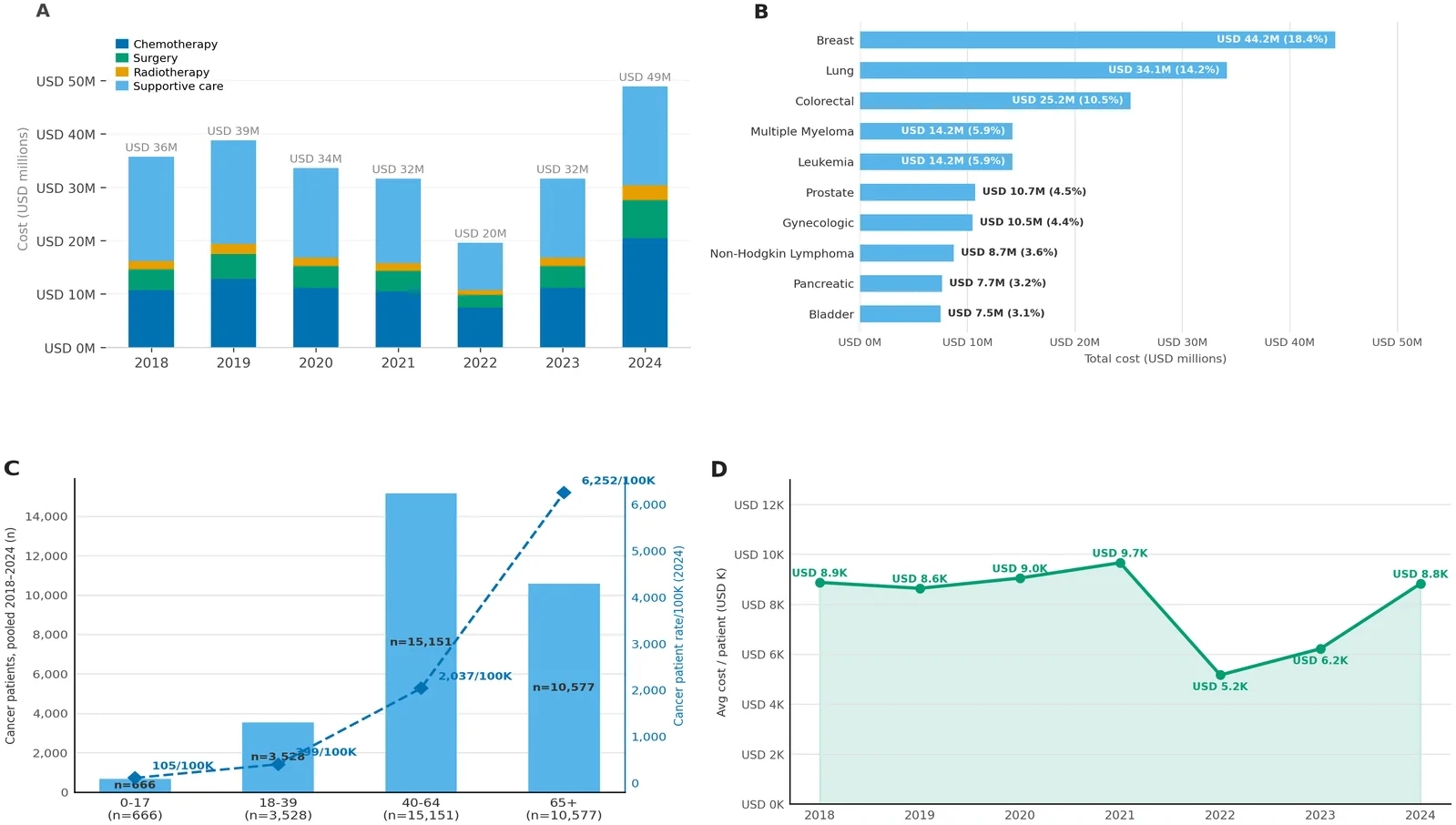
Cancer care expenditure and cost concentration (2018–2024). Panel **(A)** Annual total cancer expenditure (USD millions) stacked by care category (cancer medicines, surgery, radiotherapy, other/diagnostic) for 2018–2024. The 2022 compression (USD 19.6M) occurred while clinical volumes remained broadly stable. Panel **(B)** Total expenditure by top 10 cancer types (primary attribution, 2018–2024 combined); breast cancer leads (USD 44.2M, 18.4%), followed by lung (USD 34.1M, 14.2%). Panel **(C)** Age-stratified pooled patient counts (2018–2024, left axis, bars) and 2024 crude cancer patient rates per 100,000 insured members (right axis, diamonds), illustrating the steep age gradient in both patient volume and cancer patient rates. Panel **(D)** Average annual cancer expenditure per patient (USD), 2018–2024. Expenditure peaked at USD 9,662 in 2021; compressed to USD 5,166 in 2022 and recovered to USD 8,827 in 2024.

Total cancer care expenditure over the study period was USD 239,870,103. Annual expenditure grew from USD 35.7 million in 2018 to USD 48.9 million in 2024, with a marked compression in 2022 (USD 19.6 million) (**Figure 5A**). Average cost per patient across the study period was USD 14,435. Average annual expenditure per patient ranged from USD 5,166 in 2022 to USD 9,662 in 2021, recovering to USD 8,827 in 2024 (**Figure 5D**).

By care category, other and diagnostic services constituted the largest share of expenditure (41.8%), followed by cancer medicines (37.8%), surgery (14.5%), and radiotherapy (5.8%). This category comprises claims that could not be attributed to a single treatment modality rather than a distinct service type. Bundled inpatient episodes billed as lump-sum all-inclusive claims accounted for 78.2% of it, itemized inpatient stays 4.5%, laboratory and pathology 4.4%, diagnostic imaging 1.6%, pharmacy and supportive medication 1.2%, supplies and devices 1.1%, and physician services 1.0%, with 8.0% unclassified. Expenditure cannot be apportioned per service because the payer records a single paid amount per claim rather than per line item. Among treatment-specific categories, cancer medicines were the largest cost driver. This highlights a key asymmetry, where despite surgery being the most common modality by patient count, cancer medicines (received by a smaller proportion of patients) generated the highest treatment-specific spend, a pattern consistent with the higher cost of systemic therapy episodes within this claims environment.

By cancer type (primary attribution), breast cancer generated the largest total expenditure (USD 44.2 million, 18.4%), consistent with its prevalence dominance. Lung cancer ranked second (USD 34.1 million, 14.2%), despite ranking third in patient-level prevalence, reflecting higher per-patient treatment costs (**Figure 5B**). The top five cancer types by total cost (breast, lung, colorectal, multiple myeloma, and leukemia) collectively accounted for 55.0% of total expenditure. Age-stratified patient counts demonstrated a steep gradient, with the 40–64 and 65+ groups comprising the majority of patient volume; crude cancer patient rates per 100,000 insured members in 2024 rose markedly with age (**Figure 5C**).

**Figure 6:**
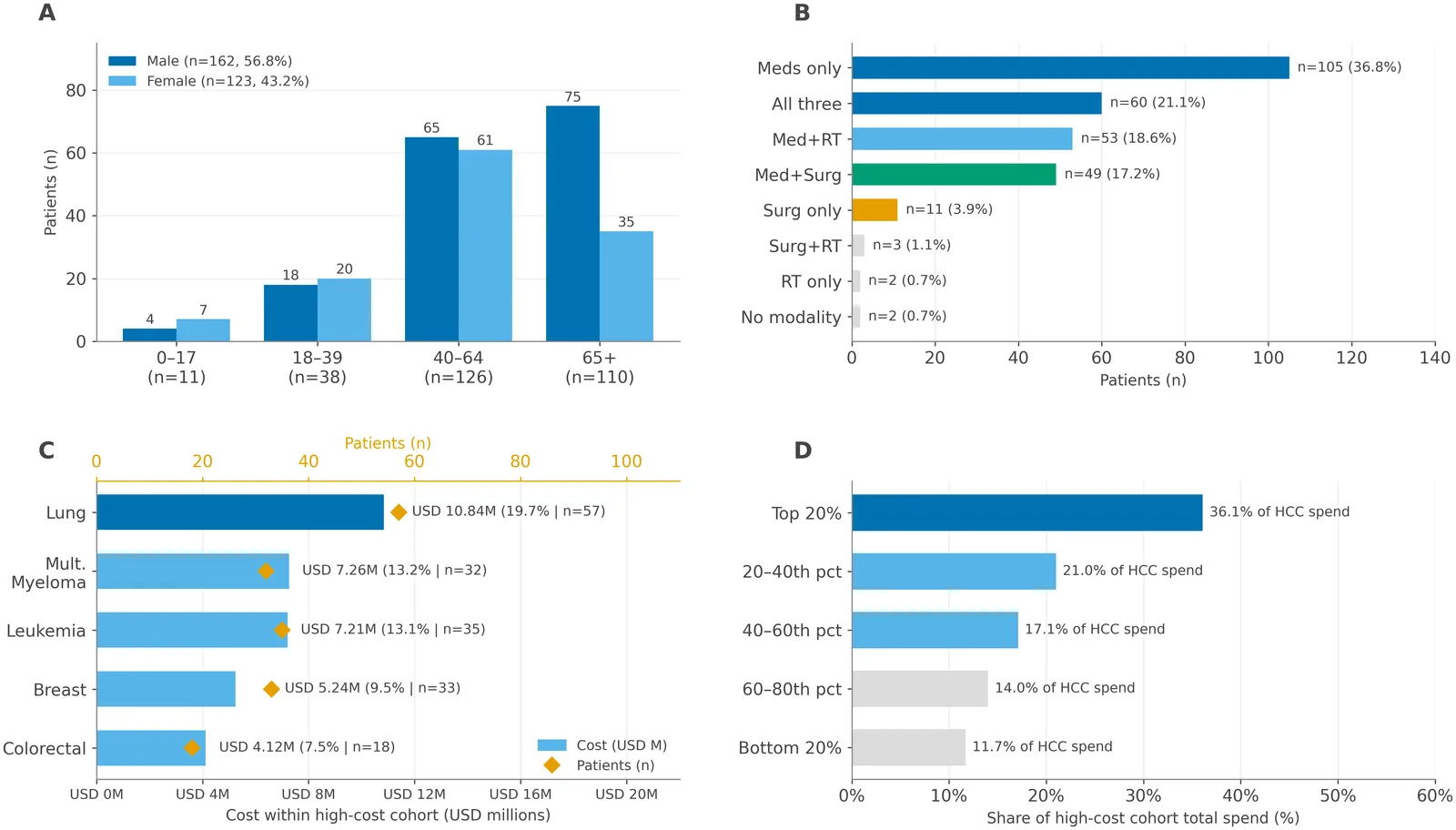
High-cost cohort characterization. High-cost cohort defined as patients incurring ≥USD 100,000 within any rolling 365-day window (n = 285; 1.7% of cohort; USD 55.1M total across the full study period). Panel **(A)** HCC patient demographics (age group and sex distribution) compared with the full cohort. Panel **(B)** Treatment modality distribution within the HCC; cancer medicines (alone or in combination) were received by most HCC patients (Cancer Medicines Only 36.8%; All Three modalities 21.1%; Medicines + Radiotherapy 18.6%; Medicines + Surgery 17.2%), indicating that systemic oncology agents feature in most high-cost episodes. Panel **(C)** Cancer-type cost distribution within the HCC; lung cancer leads (USD 10.84M, 19.7%), followed by multiple myeloma (USD 7.26M, 13.2%) and leukemia (USD 7.21M, 13.1%). Panel **(D)** Within-HCC expenditure concentration by cost quintile; the top 20% of HCC patients accounted for 36.1% of HCC total spend, while the bottom 20% held 11.7%, confirming expenditure concentration even within this already high-cost subgroup.

A high-cost cohort of 285 patients (1.7% of the full cohort) was identified as incurring USD 100,000 or more within a rolling 365-day window, together accounting for USD 55.1 million, or 23.0% of total expenditure across the full study period (**Figure 6**). Mortality within this cohort was substantially higher than in the full cohort (34.0% versus 7.2%), and the cohort was male-predominant (56.8%) in contrast to the female predominance of the full cohort. Within the HCC, the cancer type cost distribution diverged sharply from the general cohort with lung cancer leading at USD 10.84 million (19.7% of HCC spend), followed by multiple myeloma (USD 7.26 million, 13.2%) and leukemia (USD 7.21 million, 13.1%). Breast cancer, despite leading full-cohort prevalence and total expenditure, had a proportionally smaller HCC cost share, underscoring that hematological malignancies and lung cancer, which are characterized by expensive systemic agents and intensive inpatient episodes, constitute the high-cost tail. Cancer medicines dominated HCC expenditure by care category.

**Figure 7:**
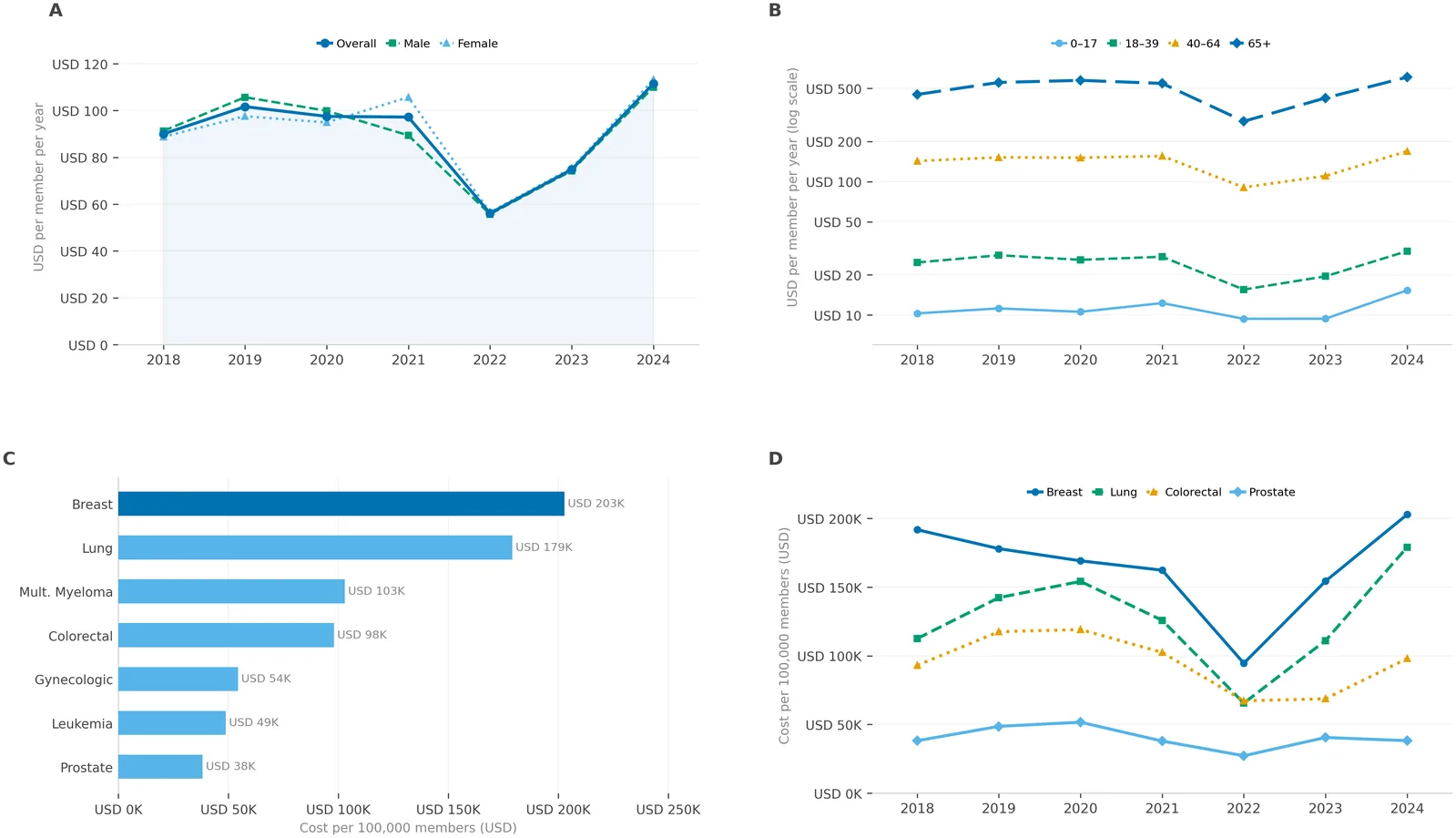
Per-member-per-year cancer expenditure (2018–2024). Panel **(A)** Overall PMPY trend by year (USD per insured member), with male and female PMPY overlaid; overall PMPY rose from USD 90.03 in 2018 to USD 111.50 in 2024, with a trough of USD 56.10 in 2022; sex-stratified PMPY tracked closely, with female PMPY modestly higher across most years. Panel **(B)** PMPY by age group (log scale), illustrating the steep per-member cost gradient; the 65+ group reached USD 606 per member in 2024, compared with USD 169 for 40–64, USD 30 for 18–39, and USD 15 for 0–17. Panel **(C)** Population burden per 100,000 insured members by top cancer types in 2024 (cost per 100,000 members, USD), showing the dominance of breast and lung cancer. Panel **(D)** Cancer-type-specific expenditure per 100,000 insured members for the four highest-burden types (breast, lung, colorectal, prostate), all showing recovery and growth from 2021 onwards, consistent with the overall population burden trajectory.

Annual cancer expenditure per insured member and its stratification are presented in **Figure 7** and **Supplementary Table 4**. Overall per-member expenditure was USD 90.03 in 2018, rising to USD 101.68 in 2019 before declining over 2020–2021 with the pandemic. Per-member expenditure compressed to USD 56.10 in 2022. Patient volumes remained broadly stable in 2022 (n = 3,796). Per-member expenditure recovered to USD 74.80 in 2023 and reached USD 111.50 in 2024, the highest value recorded in the study period.

Age stratification revealed a steep per-member cost gradient. The 65+ age group bore the highest burden, reaching USD 606 per member in 2024, compared with approximately USD 169 for the 40–64 group, USD 30 for 18–39, and USD 15 for 0–17 (**Figure 7B**). Sex-stratified per-member expenditure tracked closely between males and females. Female per-member expenditure was modestly higher across most years (**Figure 7A**). Cancer-type-specific per-member expenditure (**Figures 7C, D**) demonstrated rising per-member costs for breast, lung, colorectal, and prostate cancers from 2021 onwards, consistent with the recovery in overall per-member expenditure.

## 4 Discussion

### 4.1 Principal findings

This study provides the first seven-year, longitudinal, claims-based characterization of the economic burden of cancer care in Lebanon’s privately insured population. Three findings are of particular actuarial and policy relevance.

First, cancer expenditure is highly concentrated. A mere 1.7% of patients (the 285-patient HCC) generated 23.0% of total spend. This degree of concentration is consistent with patterns reported in high-income health systems. Studies from the United States and Canada have consistently shown that the top 1–5% of cancer patients account for 20–30% of total oncology expenditure, typically driven by late-stage disease requiring expensive systemic agents (22–25). The observation of a similar concentration in a LMIC private insurance context reinforces the case for targeted HCC management programs (30, 31), including proactive case management, utilization review for high-cost regimens, and risk-sharing arrangements. Comparison should be made with care, as those studies commonly define high-cost patients by expenditure percentile rather than by an absolute monetary threshold.

Second, surgery leads by patient volume but cancer medicines dominate by cost. This dissociation, where surgery is most common (29.3% of patients) yet cancer medicines the largest cost category (37.8% of spend), reflects the high unit cost of oncology agents relative to surgical procedures within this claims environment. As precision oncology and immunotherapy penetration grows in Lebanon, this asymmetry is likely to intensify (28). The finding has direct implications for benefit design. Formulary management, prior authorization, and step-therapy protocols for systemic agents represent higher-leverage cost controls than surgical utilization management (27).

Third, the breast-leads-prevalence/lung-leads-HCC-cost tension is a pivotal insight for actuarial planning. Breast cancer’s dominance in prevalence and total expenditure is expected and consistent with regional epidemiology (14, 15). However, within the HCC, lung cancer (19.7%) and hematological malignancies (33.9%) collectively account for 53.6% of HCC spend. These are cancers characterized by high-cost systemic regimens including targeted therapies, immunotherapy, and for eligible patients, stem cell transplantation (26). Plans pricing stop-loss cover around average cancer costs will underestimate the financial tail risk concentrated in these disease categories.

### 4.2 Rising cancer patient rates and actuarial implications

The 24.5% rise in crude cancer patient rates per 100,000 insured members persisted after age standardization, suggesting that the increase was not fully explained by changes in the age–sex composition of the insured pool. Several mechanisms may contribute. Expanding cancer screening uptake within the insured population (which tends to be more health-literate and diagnostically active than the uninsured), changing lifestyle risk factor prevalence (obesity, sedentary behavior, tobacco use), and improved diagnostic capacity at TPA-contracted hospitals (17). Lebanon’s national cancer registry documents broadly consistent trends in the general population, though direct comparison is limited by differences in case ascertainment methodology (13, 15). The compression in USD-denominated expenditure observed in 2022 most plausibly reflects the loss of USD-equivalent value of LBP-denominated cost components during the currency devaluation and Sayrafa exchange-rate transition, particularly as patient volumes remained broadly stable that year. The present design cannot formally separate exchange-rate effects from concurrent changes in utilization, provider tariffs, treatment mix or coverage terms, and this value should therefore be interpreted with caution (19, 35).

PMPY cost rose markedly with age in 2024, from USD 15 in the 0–17 group to USD 606 in the 65+ group, a 40-fold gradient with direct actuarial implications for age-banded premium structures (29, 33).

### 4.3 Limitations

Several limitations apply. First, this analysis was conducted on data from a single TPA. While this TPA is among the largest in Lebanon, its insured population may not be fully representative of all privately insured Lebanese individuals. Second, cancer diagnoses were derived from ICD-10 codes on insurance claims, which are subject to coding variability and may not reflect pathologically confirmed diagnoses. For example, disease stage was not available, precluding stage-stratified analysis. Third, the currency crisis introduces complexity into longitudinal cost comparisons, particularly for 2022–2023. Fourth, survival analyses were limited to the death flag recorded in claims data, which may undercount deaths occurring outside the insurance network. Fifth, a substantial share of claims are submitted as lump-sum all-inclusive bundles in which individual services are not itemized, so treatment modality cannot be assigned for those claims and expenditure cannot be apportioned by service. Sixth, primary cancer type was assigned from the earliest recorded diagnosis, which may not represent the tumor principally responsible for expenditure. Finally, claims data do not capture treatment intent, services obtained outside the insurance network, patient out-of-pocket expenditure, or indirect and productivity costs. The findings therefore represent private insurance claims expenditure administered by a single TPA rather than the full societal economic burden of cancer.

## 5 Conclusions

This study documents, for the first time, the longitudinal economic burden of cancer care in Lebanon’s privately insured population across a seven-year period that included a global pandemic and one of the most severe currency crises in modern history. Key findings include: 1) cancer expenditure is concentrated in a small, identifiable high-cost minority, 2) cancer medicines dominate cost despite surgery leading by patient volume, 3) lung cancer and hematological malignancies drive the high-cost tail, 4) and the rising cancer patient rate trajectory signals that this burden will grow. These findings provide an empirical actuarial foundation for Lebanese insurers, TPAs, and policymakers to design sustainable, oncology-specific benefit structures, including stop-loss provisions calibrated to the HCC cost distribution, formulary controls on high-cost systemic agents, case management programs targeting HCC-risk patients, and age-stratified premium structures informed by the steep PMPY age gradient documented here. This study characterizes expenditure and does not evaluate the effectiveness, cost-effectiveness or equity of these interventions, which will require prospective evaluation.

## Data Availability

The data analyzed in this study is subject to the following licenses/restrictions: The data supporting the findings of this study are held by a commercial third-party administrator and are not publicly available owing to commercial confidentiality. Aggregated summary statistics supporting the findings are available from the corresponding author on reasonable request. Requests to access these datasets should be directed to ss117@aub.edu.lb.
