# Supplementary material for: Economic burden of cancer care in Lebanon’s privately insured population: a seven-year retrospective cohort analysis (2018–2024)
Source: Front Oncol. 2026 Aug 25;16:1924237. doi: 10.3389/fonc.2026.1924237 (PMC13549849; doi:10.3389/fonc.2026.1924237)
Supplement: Supplementary file 1 [file Table1.docx]

**SUPPLEMENTARY TABLES**

**Supplementary Table S1. Full ICD-10 Cancer Taxonomy — All 69 Categories (2018–2024)**

| **Rank** | **Cancer Type (ICD-10)** | **Cancer-Level n** | **Cancer Level %** | **Unique Patients n** | **Patient Level %** | **Total Cost (USD)** |
| --- | --- | --- | --- | --- | --- | --- |
| 1 | Breast | 4,696 | 19.70% | 4,538 | 27.3% | $44,237,204 |
| 2 | Lung | 1,401 | 5.88% | 1,249 | 7.5% | $34,147,567 |
| 3 | Prostate | 1,387 | 5.82% | 1,312 | 7.9% | $10,726,425 |
| 4 | Non-Hodgkin Lymphoma | 1,234 | 5.18% | 1,009 | 6.1% | $8,719,808 |
| 5 | Colorectal | 1,106 | 4.64% | 1,074 | 6.5% | $25,171,512 |
| 6 | Bladder | 961 | 4.03% | 833 | 5.0% | $7,509,759 |
| 7 | Gynecologic | 746 | 3.13% | 637 | 3.8% | $10,465,606 |
| 8 | Leukemia | 633 | 2.66% | 456 | 2.7% | $14,176,905 |
| 9 | Skin (Non-Melanoma) | 555 | 2.33% | 555 | 3.3% | $1,122,128 |
| 10 | Secondary Other Sites | 550 | 2.31% | 56 | 0.3% | $857,569 |
| 11 | Brain & CNS | 543 | 2.28% | 386 | 2.3% | $7,347,958 |
| 12 | Thyroid | 486 | 2.04% | 457 | 2.7% | $2,727,805 |
| 13 | Multiple Myeloma | 476 | 2.00% | 410 | 2.5% | $14,189,975 |
| 14 | Secondary Respiratory/Digestive | 460 | 1.93% | 46 | 0.3% | $579,092 |
| 15 | Other Hematopoietic | 446 | 1.87% | 392 | 2.4% | $169,800 |
| 16 | Head & Neck | 444 | 1.86% | 442 | 2.7% | $4,304,143 |
| 17 | Hodgkin Lymphoma | 429 | 1.80% | 370 | 2.2% | $4,853,541 |
| 18 | Ill-defined Sites | 387 | 1.62% | 243 | 1.5% | $78,417 |
| 19 | Pancreatic | 369 | 1.55% | 307 | 1.8% | $7,655,251 |
| 20 | Unspecified Site | 367 | 1.54% | 151 | 0.9% | $407,257 |
| 21 | Myeloproliferative | 351 | 1.47% | 351 | 2.1% | $276,731 |
| 22 | In Situ - Other | 340 | 1.43% | 340 | 2.0% | $165,408 |
| 23 | Uncertain - Urinary | 330 | 1.38% | 330 | 2.0% | $537,453 |
| 24 | Kidney | 315 | 1.32% | 315 | 1.9% | $3,817,314 |
| 25 | Soft Tissue & Connective | 314 | 1.32% | 314 | 1.9% | $2,462,383 |
| 26 | Lymph Node (Secondary) | 291 | 1.22% | 291 | 1.8% | $980,330 |
| 27 | In Situ - Breast | 270 | 1.13% | 270 | 1.6% | $96,887 |
| 28 | In Situ - Digestive | 246 | 1.03% | 246 | 1.5% | $75,889 |
| 29 | Bone | 221 | 0.93% | 221 | 1.3% | $3,325,218 |
| 30 | Stomach | 216 | 0.91% | 216 | 1.3% | $4,606,496 |
| 31 | Liver | 216 | 0.91% | 216 | 1.3% | $5,412,264 |
| 32 | Testis | 190 | 0.80% | 190 | 1.1% | $918,103 |
| 33 | In Situ - Melanoma | 162 | 0.68% | 162 | 1.0% | $439,806 |
| 34 | Gallbladder & Biliary | 152 | 0.64% | 152 | 0.9% | $2,509,927 |
| 35 | In Situ - Other Genital | 152 | 0.64% | 152 | 0.9% | $111,812 |
| 36 | Carcinoid Tumor | 149 | 0.63% | 149 | 0.9% | $149,550 |
| 37 | Uncertain - Digestive | 143 | 0.60% | 143 | 0.9% | $191,998 |
| 38 | Uncertain - Female Genital | 141 | 0.59% | 141 | 0.8% | $372,021 |
| 39 | Uncertain - Other Sites | 131 | 0.55% | 131 | 0.8% | $274,878 |
| 40 | Uncertain - Respiratory | 129 | 0.54% | 129 | 0.8% | $279,634 |
| 41 | In Situ - Skin | 125 | 0.52% | 125 | 0.8% | $90,364 |
| 42 | Myelodysplastic Syndrome | 124 | 0.52% | 124 | 0.7% | $1,255,874 |
| 43 | Other Endocrine | 104 | 0.44% | 104 | 0.6% | $238,166 |
| 44 | Uncertain - Male Genital | 99 | 0.42% | 99 | 0.6% | $147,047 |
| 45 | Polycythemia Vera | 98 | 0.41% | 98 | 0.6% | $92,581 |
| 46 | Melanoma | 89 | 0.37% | 89 | 0.5% | $2,177,438 |
| 47 | Thymus & Heart | 88 | 0.37% | 88 | 0.5% | $772,697 |
| 48 | In Situ - Respiratory | 81 | 0.34% | 81 | 0.5% | $237,866 |
| 49 | Uncertain - Endocrine | 78 | 0.33% | 78 | 0.5% | $306,315 |
| 50 | Small Intestine | 77 | 0.32% | 77 | 0.5% | $1,855,431 |
| 51 | Esophagus | 77 | 0.32% | 77 | 0.5% | $1,958,724 |
| 52 | Kaposi Sarcoma | 77 | 0.32% | 77 | 0.5% | $314,691 |
| 53 | Adrenal | 73 | 0.31% | 73 | 0.4% | $355,745 |
| 54 | Unknown Primary | 67 | 0.28% | 67 | 0.4% | $1,233,752 |
| 55 | Other Digestive | 61 | 0.26% | 61 | 0.4% | $272,002 |
| 56 | Other Urinary | 59 | 0.25% | 59 | 0.4% | $62,228 |
| 57 | Uncertain - Brain/CNS | 57 | 0.24% | 57 | 0.3% | $289,700 |
| 58 | Ureter | 47 | 0.20% | 47 | 0.3% | $347,244 |
| 59 | Eye | 37 | 0.16% | 37 | 0.2% | $194,947 |
| 60 | In Situ - Cervix | 33 | 0.14% | 33 | 0.2% | $72,762 |
| 61 | Immunoproliferative | 30 | 0.13% | 30 | 0.2% | $117,483 |
| 62 | Secondary Neuroendocrine | 28 | 0.12% | 28 | 0.2% | $8,600 |
| 63 | Penis | 19 | 0.08% | 19 | 0.1% | $54,334 |
| 64 | Other Male Genital | 18 | 0.08% | 18 | 0.1% | $5,347 |
| 65 | Merkel Cell Carcinoma | 16 | 0.07% | 16 | 0.1% | $43,552 |
| 66 | Mesothelioma | 15 | 0.06% | 15 | 0.1% | $263,145 |
| 67 | Trachea | 11 | 0.05% | 11 | 0.1% | $355,060 |
| 68 | Other Respiratory | 7 | 0.03% | 7 | 0.0% | $3,924 |
| 69 | Uncertain - Meninges | 6 | 0.03% | 6 | 0.0% | $48,770 |

*†Unknown Primary (n = 67): Under the cancer-level framework, this count reflects patients with an Unknown Primary ICD-10 code anywhere in their all-time diagnosis history (n = 67). This figure is lower than the former primary-attribution count (n = 184) because patients initially coded as Unknown Primary commonly receive a more specific cancer diagnosis in subsequent claims, causing them to be reclassified under that diagnosis type in the cancer-level framework.*

**Supplementary Table S2. Characteristics of the High-Cost Cohort (≥USD 100,000 per Rolling 365-Day Window)**

| **Characteristic** | **n** | **%** |
| --- | --- | --- |
| ***Cohort Overview*** | | |
| High-cost patients (≥USD 100K / rolling year) | 285 | 1.7% of cancer cohort |
| Total expenditure (USD) | $55,138,674 | — |
| Mean total cost per patient (USD) | $193,469 | — |
| Deceased at last follow-up | 97 | 34.0% |
| Cancer Medicines expenditure (USD) | $28,268,946 | — |
| Surgery expenditure (USD) | $2,157,533 | — |
| Radiotherapy expenditure (USD) | $1,015,196 | — |
| ***Age Distribution*** | | |
| 0-17 | 11 | 3.9% |
| 18-39 | 38 | 13.3% |
| 40-64 | 126 | 44.2% |
| 65+ | 110 | 38.6% |
| ***Sex*** | | |
| Male | 162 | 56.8% |
| Female | 123 | 43.2% |
| ***Treatment Modality Combinations*** | | |
| Cancer Medicines Only | 105 | 36.8% |
| Surgery Only | 11 | 3.9% |
| Radiotherapy Only | 2 | 0.7% |
| Medicines + Surgery | 49 | 17.2% |
| Medicines + Radiotherapy | 53 | 18.6% |
| Surgery + Radiotherapy | 3 | 1.1% |
| All Three Modalities | 60 | 21.1% |
| No modality recorded | 2 | 0.7% |
| ***Top 10 Cancer Types in High-Cost Cohort*** | | |
| Lung | 57 | 20.0% |
| Multiple Myeloma | 32 | 11.2% |
| Leukemia | 35 | 12.3% |
| Breast | 33 | 11.6% |
| Colorectal | 18 | 6.3% |
| Non-Hodgkin Lymphoma | 12 | 4.2% |
| Prostate | 8 | 2.8% |
| Pancreatic | 9 | 3.2% |
| Bladder | 6 | 2.1% |
| Head & Neck | 6 | 2.1% |
| ***Cost Concentration (Quintiles within High-Cost Cohort)*** | | |
| Top 20% | 57 | 36.1% |
| 20-40% | 57 | 21.0% |
| 40-60% | 57 | 17.1% |
| 60-80% | 57 | 14.0% |
| Bottom 20% | 57 | 11.7% |

**Supplementary Table S3. Claims-Based Cancer Patient Rates and Cost Trends (2018–2024)**

| **Year** | **Insured Population** | **Cancer Patients (Annual)** | **Rate per 100,000** | **Total Cancer Cost (USD)** | **PMPM (USD)** | **PMPY (USD)** |
| --- | --- | --- | --- | --- | --- | --- |
| 2018 | 396,230 | 4,020 | 1014.6 | $35,671,957 | $7.50 | $90.03 |
| 2019 | 381,716 | 4,493 | 1177.1 | $38,811,554 | $8.47 | $101.68 |
| 2020 | 345,039 | 3,718 | 1077.6 | $33,643,064 | $8.13 | $97.51 |
| 2021 | 325,387 | 3,274 | 1006.2 | $31,632,970 | $8.10 | $97.22 |
| 2022 | 349,565 | 3,796 | 1085.9 | $19,610,669 | $4.68 | $56.10 |
| 2023 | 421,945 | 5,077 | 1203.2 | $31,560,408 | $6.23 | $74.80 |
| 2024 | 438,911 | 5,544 | 1263.1 | $48,939,481 | $9.29 | $111.50 |

**Supplementary Table S4. Per-Member-Per-Year (PMPY) Cancer Cost by Type — Top 15 (2018–2024)**

| **Cancer Type** | **2018** | **2019** | **2020** | **2021** | **2022** | **2023** | **2024** | **Mean PMPY** | **Rank** |
| --- | --- | --- | --- | --- | --- | --- | --- | --- | --- |
| Breast | $15.86 | $15.07 | $13.88 | $14.31 | $7.61 | $12.72 | $18.30 | **$13.96** | 1 |
| Lung | $7.53 | $8.31 | $9.10 | $8.42 | $4.20 | $6.62 | $10.90 | **$7.87** | 2 |
| Leukemia | $5.85 | $8.19 | $6.58 | $5.29 | $3.06 | $5.11 | $7.71 | **$5.97** | 3 |
| Colorectal | $5.45 | $7.52 | $6.37 | $5.75 | $3.60 | $4.10 | $6.17 | **$5.57** | 4 |
| Multiple Myeloma | $4.46 | $4.16 | $3.58 | $3.56 | $2.19 | $4.78 | $8.53 | **$4.47** | 5 |
| Secondary Other Sites | $4.03 | $4.69 | $4.89 | $4.14 | $1.72 | $2.50 | $3.95 | **$3.70** | 6 |
| Prostate | $3.18 | $4.44 | $4.62 | $3.80 | $2.36 | $3.46 | $3.86 | **$3.67** | 7 |
| Non-Hodgkin Lymphoma | $3.09 | $4.15 | $3.92 | $3.83 | $2.65 | $2.58 | $4.10 | **$3.48** | 8 |
| Brain & CNS | $4.39 | $3.45 | $3.64 | $3.46 | $2.26 | $2.11 | $2.80 | **$3.16** | 9 |
| Gynecologic | $3.40 | $3.22 | $2.97 | $3.73 | $1.97 | $2.32 | $4.24 | **$3.12** | 10 |
| Secondary Respiratory/Digestive | $2.89 | $3.13 | $3.65 | $4.22 | $2.21 | $2.23 | $3.01 | **$3.05** | 11 |
| Pancreatic | $2.68 | $3.20 | $2.17 | $3.23 | $2.25 | $1.72 | $1.94 | **$2.46** | 12 |
| Bladder | $2.79 | $2.60 | $2.40 | $1.98 | $1.53 | $2.07 | $3.12 | **$2.36** | 13 |
| Hodgkin Lymphoma | $1.58 | $1.90 | $1.81 | $1.43 | $0.97 | $1.38 | $2.29 | **$1.62** | 14 |
| Liver | $1.49 | $1.48 | $1.27 | $1.39 | $1.03 | $0.94 | $1.16 | **$1.25** | 15 |
